# Porous NiTi Particle Dispersed Mg-Zn-Ca Bulk Metallic Glass Matrix Composites

**DOI:** 10.3390/ma11101959

**Published:** 2018-10-12

**Authors:** Wei Guo, Hidemi Kato, Shulin Lü, Shusen Wu

**Affiliations:** 1 State Key Lab of Materials Processing and Die & Mould Technology, School of Materials Science and Engineering, Huazhong University of Science and Technology, 1037 Luoyu Road, Wuhan 430074, China; ssw636@hust.edu.cn; 2 Institute for Materials Research, Tohoku University, Sendai 980-8577, Japan; hikato@imr.tohoku.ac.jp

**Keywords:** amorphous materials, composite materials, mechanical property, corrosion

## Abstract

Even though the Mg-based bulk metallic glasses (BMGs) have shown superior anti-corrosion properties compared with their crystalline counterparts, the brittleness of them limits the widespread application of these materials. In the present study, we have firstly introduced porous NiTi shape memory alloy particles into an Mg-Zn-Ca BMG by the direct adding method. This composite showed both improved compressive strength and corrosion resistance in Hank’s solutions than its monolithic glassy counterpart. The NiTi dispersoids among the matrix were likely to hinder the main shear band propagation, and also acted as the corrosion barriers. Furthermore, the porous nature of present added particle could further increase the interface areas, which should enhance the reinforcing effects compared with solid ones. This low-cost, high-anticorrosive composite was a good candidate as an engineering material.

## 1. Introduction

Magnesium (Mg) alloys have received tremendous research interest for their great potentials as bio-implant materials [1,2,3]. However, conventional Mg alloys can hardly meet the requirements for such applications due to several intrinsic deficiencies. Firstly, the mechanical strength of conventional Mg alloys is not high enough. In corrosive environment, the strength would even deteriorate gradually [4]. Secondly, non-uniform corrosion (such as pitting corrosion) imposes additional risk of disintegration for the Mg alloys [5]. Thirdly, the corrosion rate of Mg alloys is much faster than the bone healing rate [6].

Regarding these disadvantages, Mg-based bulk metallic glasses (BMGs) have been considered as a more promising candidate since they generally possess superior mechanical properties (e.g., ~40% higher specific strength compared with conventional crystalline Mg alloys), impressive corrosion resistance and relatively more uniform corrosion behaviors than conventional Mg alloys [7,8,9]. Among Mg-based BMGs, Mg-Zn-Ca BMGs is the very branch with remarkable attraction for bio-applications because of their low density (~2.0 g/cm^3^) and low Young’s modulus (25–45 GPa), which is close to the modulus of human bones (10–40 GPa) [10]. Nevertheless, the brittleness of Mg-Zn-Ca BMGs at room temperature significantly restricts them from practical applications [11]. As inspired by our recent achievement of improved mechanical properties in the ex-situ porous NiTi particle reinforced Mg-Cu-Gd-Ag bulk metallic glass matrix composites (BMGMCs) [12], we make attempts in introducing porous NiTi particles into Mg-Zn-Ca glassy matrix in this study. It is noted that NiTi is also a good candidate for bio-applications owing to its excellent mechanical properties and corrosion resistance [13]. The microstructure, mechanical properties, and corrosion properties of the porous NiTi particle dispersed Mg-Zn-Ca bulk BMGMC are investigated in detail to evaluate its potentials as biomaterials.

In early work from Ma et al., the best glass-former in Mg-Zn-Ca system was identified at the composition of Mg_67_Zn_29_Ca_4_ (at. %), with the critical rod size approaching five mm in diameter [14]. Therefore, this composition was selected here as the glass matrix. The detailed fabrication process of porous NiTi particles can be found somewhere else [12].

## 2. Materials and Methods 

A mixture of pure Mg, Zn and Ca was achieved by induction heating under a helium atmosphere at 1073 K for 2 min. During the melting, porous NiTi particles (average size of ~30 μm) were doped into the Mg-Zn-Ca melt with a volume fraction of 3%. Meanwhile, mechanical stirring was exerted to obtain a better mixture between the particles and melt. Since the melting temperature was much lower than the melting point of NiTi [13] and the reactivity between NiTi and the melt is quite limited [12], the porous NiTi particles did not melt or dissolve during fabrication process. For comparison, a monolithic Mg_67_Zn_29_Ca_4_ alloy (base sample) was also prepared. Afterward, the Mg_67_Zn_29_Ca_4_ and Mg_67_Zn_29_Ca_4_/NiTi master ingots were inductively remelt in a quartz tube and injected into a copper mold cavity with the diameter of 2 mm to obtain the final samples.

The microstructures of investigated samples were examined by X-ray diffraction (XRD; Bruker D8 Advance, Yokohama-shi, Japan) with Cu Kα radiation and scanning electron microscopy with attached energy-dispersive X-ray spectrometry (SEM-EDX; Carl Zeiss Ultra 55 with Bruker AXS, Oberkochen, Germany). Uniaxial compression tests were performed at a strain rate of 5 × 10^−4^·s^−1^ at room temperature in an Instron 4204 machine (Boston, MA, USA). Compression specimens with the height of 4 mm and diameter of 2 mm were cut in parallel and carefully polished to ensure the end flatness. At least four samples were used in compression test to confirm reproducibility. The corrosion behavior was evaluated by electrochemical measurements. The samples for corrosion tests were coated with epoxy resin, except for a measurement area of 3.14 mm^2^. The electrolytes used were Hank’s solution, which was utilized to simulate the circumstance of human body [15]. Electrochemical measurements were conducted in a three-electrode cell using a platinum counter electrode and a saturated calomel reference electrode (SCE). Potentiodynamic polarization curves were measured with a potential sweep rate of 50 mV/min in Hank’s solution open to air at 298 K after immersing the samples for about 10 min when the open-circuit potentials became steady. At least four samples were used in electrochemical tests to confirm the reproducibility.

## 3. Results and Discussion

**Figure 1:**
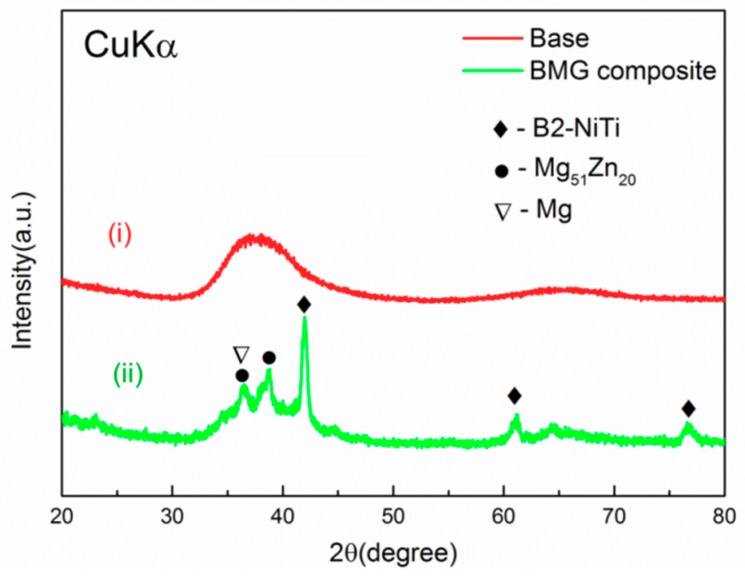
XRD patterns for both (**i**) base alloy and (**ii**) composite with 3 vol.% porous NiTi addition.

Figure 1 shows XRD patterns of base alloy (Mg_67_Zn_29_Ca_4_) and Mg_67_Zn_29_Ca_4_/NiTi BMG composite containing 3 vol. % NiTi dispersoids. As we can see, the base alloy has a broad peak of single glassy structure. Distinctive peaks from B2-NiTi phase and several weak peaks from Mg and Mg_51_Zn_20_ phases are visible in the pattern of Mg_67_Zn_29_Ca_4_/NiTi composite. These results indicate that the addition of 3 vol. % NiTi particles is sufficient to degrade the glass-forming ability (GFA) of Mg_67_Zn_29_Ca_4_, agreeing well with previous studies [16]. Apparently, the incorporation of NiTi particles into the Mg-Zn-Ca BMG was successful, just as expected.

**Figure 2:**
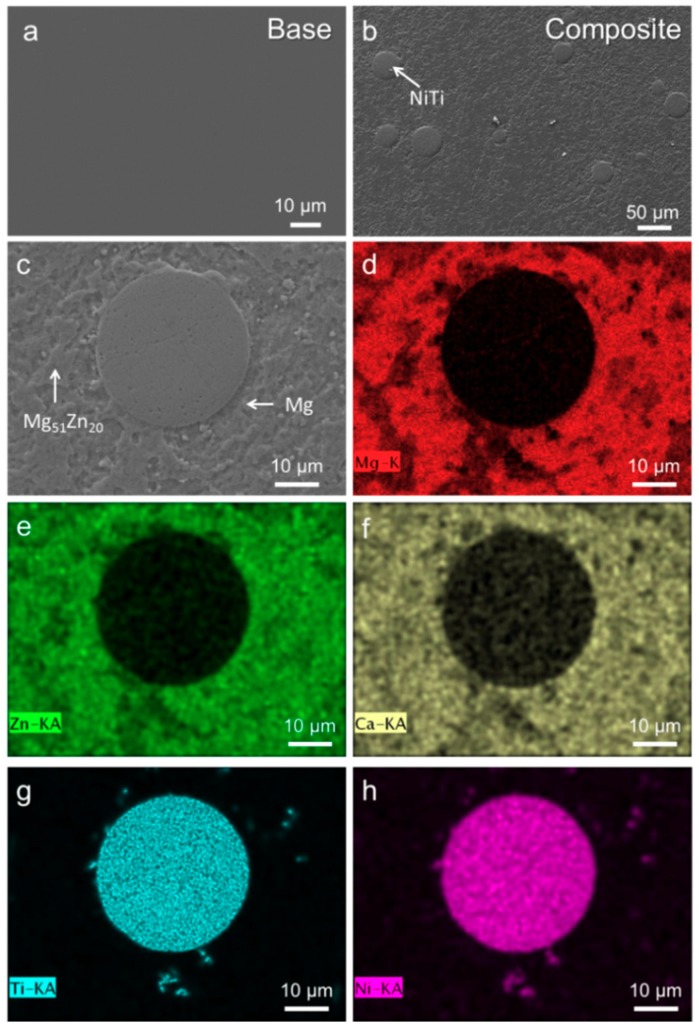
SEM images for (**a**) base alloy; (**b**,**c**) BMG composite at different magnifications; (**d**–**h**) EDX mapping taken from (**c**) ((**d**): Mg; (**e**): Zn; (**f**): Ca; (**g**): Ti; and (**h**): Ni).

Figure 2a illustrates the microstructure of base alloy. There is no distinct crystalline contrast noticed over the entire cross-section of this sample. In the SEM images of Mg_67_Zn_29_Ca_4_/NiTi BMG composite in Figure 2b,c, the dispersion of NiTi particles among the matrix is clearly revealed. To further investigate the microstructure of this composite, relevant EDX element mappings were acquired, as shown in Figure 2d–h (d: Mg, e: Zn, f: Ca, g: Ti, h: Ni). Mg-rich and Mg-poor areas can be easily distinguished from these mappings, which were determined as Mg and Mg_51_Zn_20_ phases by EDX point analysis (not shown here), respectively. In addition, the porous structure of NiTi particles is also confirmed from the mapping results of Ni and Ti (Figure 2g,h). Mg, Zn and Ca from the matrix are found to permeate into the porous NiTi particles because the composition located at the particle are detected to be Ni_39_Ti_40_Mg_15_Zn_5_Ca_1_ (at. %), indicating a good wetting between them.

**Figure 3:**
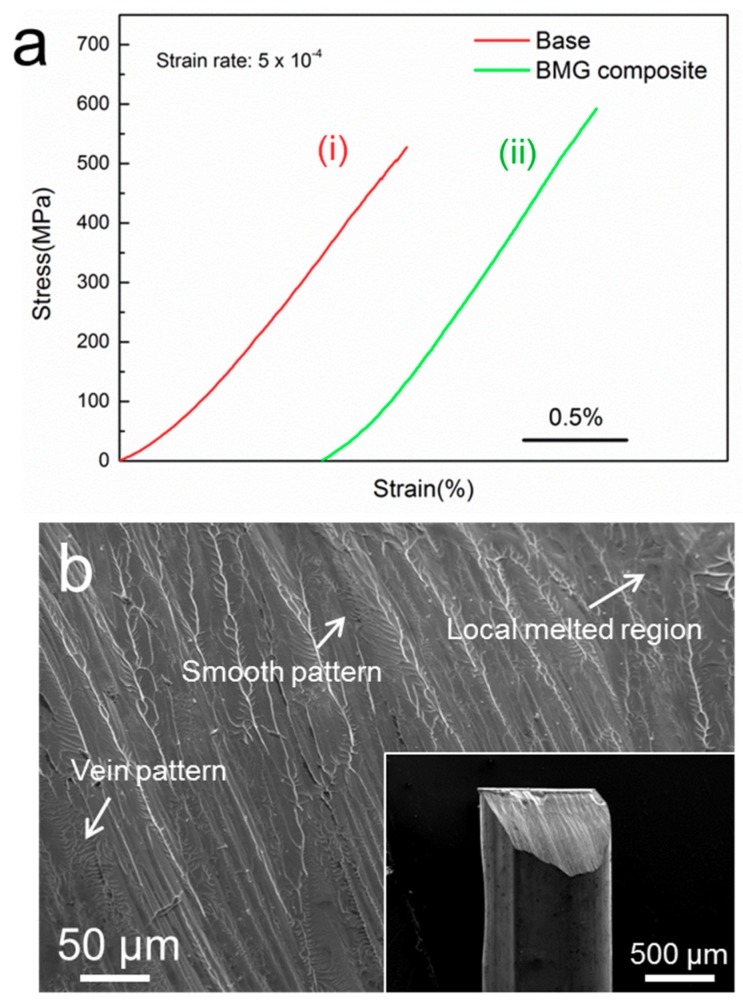
(**a**) Compressive stress-strain curves for (**i**) base and (**ii**) BMG composite; and (**b**) SEM images of fracture surfaces for the composite (image at low magnification is inserted).

The uniaxial compressive engineering stress-strain curves of monolithic Mg_67_Zn_29_Ca_4_ and Mg_67_Zn_29_Ca_4_/NiTi composite are shown in Figure 3a. The base alloy exhibited only a linear elastic deformation before fracture at the stress of ~527 ± 20 MPa. This fracture stress is comparable to the values reported before [11]. The composite fractured at a higher stress of ~592 ± 22 MPa, which, specifically, is 12% higher than that of its monolithic base counterpart. This improved strength may be associated with the presence of NiTi particles and interactions between the particles and matrix, in accordance with our previous studies of another Mg-based BMGMC [12,17]. However, due to the limited volume faction of NiTi and the partially crystallized matrix, no plastic deformation was observed in this composite. 

Since the base sample fractured into small pieces in compression test, fracture surfaces analysis is extremely difficult for this sample. However, large fractured fragments were obtained from the composite due to the improved mechanical properties (the inset in Figure 3b), which enabled the observation of its fracture morphology, with a typical image displayed in Figure 3b. A combination of smooth region, vein pattern region, and local melted region can be observed from the fracture surface, indicating that localized melting occurred during the final fracture along the shear band. In the compression test, numerous elastic energy stored upon deformation are released by shear band growth and local heating, which leads to local temperature increases (sometimes up to the melting point of the glass) [18]. The smooth region results from shear sliding, suggesting that shear propagates in an unimpeded mode over large distance, contributing to the brittleness. The vein pattern is typical to glassy alloys and indicates a certain degree of plasticity. The plastic porous B2-NiTi particle can hinder the rapid propagation of main shear bands, increasing the area of vein pattern and decreasing the area of the smooth region. Thus, the fracture of the composites is impeded and subsequently improves the fracture strength. Moreover, the porous nature of the particle can induce two kinds of reinforcing areas, large scale between two particles and small scale inside one particle, which benefits the reinforcing effects because of the complicated stress field. In addition, the stress-induced martensitic transformation from B2-NiTi to B19’-NiTi had not been detected in our Mg_67_Zn_29_Ca_4_/NiTi composite, which might be attributable for the low strength of the matrix and the limited volume fraction of NiTi dispersoids. 

**Figure 4:**
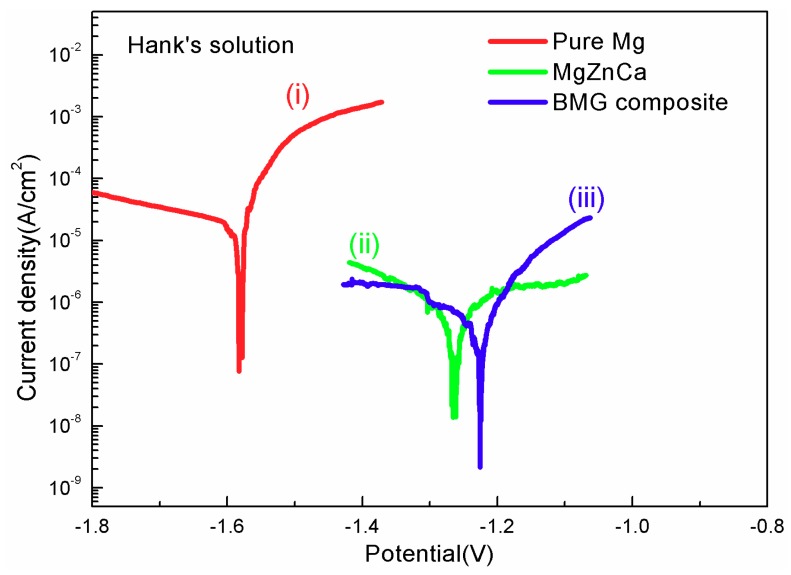
Potentiodynamic polarization curves of (**i**) pure magnesium; (**ii**) base alloy and (**iii**) BMG composite.

Typical potentiodynamic polarization curves obtained from the base alloy and composite in Hank’s solution open to air at room temperature are shown in Figure 4. A pure magnesium sample was also tested under the same condition for comparison. It can be seen that pure magnesium showed quick dissolution at the anodic side. In contrast, the base BMG showed higher positive corrosion potentials and lower corrosion current densities than those of pure magnesium, which indicates the chemical homogeneous nature of the amorphous alloy could significantly improve corrosion resistance [9]. As for the Mg_67_Zn_29_Ca_4_/NiTi composite, an even lower current density and a higher positive potential were achieved, revealing its higher corrosion resistance than the monolithic base counterpart. As discussed before, the matrix of the composite crystallized because of the addition of porous NiTi particles, and thus, the corrosion behavior of the composites should be easier than the base sample [19]. However, the existence of the NiTi dispersoids with outstanding corrosion resistance among the matrix is suggested to be strong corrosion barriers against the corrosion propagation and to decrease the corrosion rate efficiently. When the corrosion is developing, the NiTi phase may be able to prevent corrosion from spreading from one a grain to another directly across the matrix. The NiTi could slow down the corrosion process either by spreading laterally or by progressing deep into the alloy. Furthermore, the porous nature of added particles could provide more interface areas compared with solid particles, which should further enhance the barrier effects in the corrosion test.

The addition of porous NiTi has degraded the glass forming ability of the matrix seriously, thus fully amorphous matrix could not be prepared in this study and the spontaneous passivation behavior in electrochemical test disappears for the composite. Our future work will focus on the optimization of chemical composition to introduce a completely glassy matrix for the Mg-base BMG composites and a more systematic investigation of corrosion properties for them may be, in this section, be divided by subheadings. It should provide a concise and precise description of the experimental results, their interpretation as well as the experimental conclusions that can be drawn.

## 4. Conclusions

A novel porous NiTi particle reinforced Mg-Zn-Ca bulk metallic glass matrix composite has been successfully developed by ex-situ direct adding method. The dispersion of porous NiTi particles can effectively increase the compressive strength but degrade the glass-forming ability for the glassy matrix. The composite exhibits higher corrosion resistance than pure magnesium and its monolithic glassy counterpart because of the corrosion barrier effects from NiTi dispersoids. This low-cost Mg-Zn-Ca BMG composite combining good mechanical and corrosion properties is considered to be a good candidate as engineering biomaterials.
